# The system-wide effects of dispatch, response and operational performance on emergency medical services during Covid-19

**DOI:** 10.1057/s41599-022-01405-z

**Published:** 2022-11-18

**Authors:** Ivan L. Pitt

**Affiliations:** Independent Academic, New York, NY USA

**Keywords:** Operational research, Health humanities

## Abstract

In this paper, we analyze the Fire Department of New York City’s pre-hospital emergency medical services dispatch data for the period of March 20, 2019–June 13, 2019, and the corresponding Covid lockdown period of March 20, 2020–June 13, 2020. A fixed effects negative binomial model is used to estimate the heterogeneity effects of average ambulance travel or response times on the daily volume of emergency calls, year, day of the week, dispatcher-assigned medical emergency call type, priority rank, ambulance crew response, borough and an offset for missing calls. We also address the limitations of other non-parametric Covid studies or parametric studies that did not properly account for over-dispersion. When our model is estimated and corrected for clustered standard errors, fixed effects, and over-dispersion, we found that Wednesday was the only day of the week that was most likely to increase travel response time with an odd ratio of 6.91%. All grouped call types that were categorized showed significant declines in average travel time, except for call types designated as allergy and an odds ratio of 21.81%. When compared to Manhattan, Staten Island ambulance response times increased with an odds ratio of 19.05% while the Bronx showed a significant decline with an odds ratio of 31.92% advanced life support (ALS) and BLS ambulances showed the biggest declines in travel time with the exception of BLS assigned ambulance types and emergency priority rank of 6. Surprisingly, in terms of capacity utilization, the dispatch system was not as overwhelmed as previously predicted as emergency call volume declined by 8.83% year over year.

## Introduction

The Fire Department of New York (FDNY) is one of the largest fire departments in the United States. In addition to providing fire protection and other public safety functions, they handle emergency medical system (EMS) 911 calls for medical and non-medical emergencies and provide pre-hospital emergency medical care and transport for the residents of the five boroughs (Bronx, Brooklyn, Manhattan, Queens, and Staten Island) in New York City. Dispatchers, emergency medical technicians (EMTs), paramedics, and firefighters may be among the various types of certified first responders (CFRs) that respond to medical emergencies and some non-medical related emergencies in the city.

In responding to thousands of life-threatening and non-life-threatening medical calls each month, each activity involved in the EMS call process—from the beginning 911 call initiation to a final resolution that ends with the EMT’s hospital departure—is timed and recorded and used as *FDNY Citywide Performance Indicators*. Call volume is an aggregation of the number of FDNY emergency calls that occurred within a fixed time period such as each hour, day, or week. The daily call volume—computer-aided dispatch (CAD) incidents—received by FDNY 911 emergency call centers are often modeled as counts, given the nature of its discreteness, nonlinear and non-negative values.

The modeling of EMS call center data was often non-parametric or the wrong statistical distribution was used with dubious results even before the Covid-19 pandemic appeared. For example, in their literature review of the few studies estimating EMS response times models before the pandemic, Matteson et al. (2011) found that they were problematic and rudimentary because they were often based on *Gaussian linear models* that were in conflict with Poisson distribution theory and some of its special cases.

In a more recent study, Zhou (2016) found that ‘the current industry practice to predict ambulance demand is crude, while the few methods in prior literature—for example, the works cited in Henderson (2009)—are barely more accurate.’ In addressing, the challenges in predicting ambulance demand, Zhou (2016) proposed three flexible estimation methods based on ‘time-varying Gaussian mixture models (GMM)’, ‘kernel density (stKDE)’, and ‘kernel warping’ (WARP) theory for what they call ‘spatio-temporal predictions’ for two cities. Gaussian distribution mixture models proposed by Scrucca et al. (2016) were also used to improve the accuracy of their *k*-means clustering algorithm with a mean and covariance instead of a distance-based one.

Based on logLik estimates (sometimes using ranges and other times not), Zhou (2016) GMM estimation method is preferred for Toronto, but their WARP method is preferred for Melbourne. For example in their paper, the GMM estimation has the smallest logLik with a range of GMM = 6.07−6.15 and would suggest that it is the better model when compared to stKDE = 6.10−6.11 (given as a range), MEDIC = 8.64 and naiveKDE = 6.87 at least for the Toronto data.

There is no logLik estimate for their WARP method for Toronto. Their WARP model with logLik = 7.53−7.56 is smaller than the second-place GMM method with logLik = 7.87−7.96 and seems to work for Melbourne, but there is no logLik estimate for the stKDE method. Furthermore, their methods showed mixed results and it was (confusingly) hard to make a statistical decision on which of their three methods should be used for implementation that would be stable and reproducible over time. One of the major flaws in clustering analysis is that an attempt is made to group the association between variables—that may share some similarity but their association may be unknown—by distance and the clusters are arbitrarily defined by ‘*k*.’ This is akin to looking for some sort of a pattern within the data, but the researchers are not sure what the pattern is; and the results can, therefore, produce misleading findings and interpretations.

More recent Covid-19 academic studies were just as problematic as earlier works. For example, Prezant et al. (2020) developed a ‘longitudinal’ non-parametric analysis using FDNY’s EMS data in similar time periods to our study that was limited to a (pairwise) cross-tabulation of contingency tables, and ambulance crew data was omitted included in their analysis. The underlying Poisson distribution was not utilized in this analysis which weakened another significant contribution to Covid studies. A recent longitudinal study using an unbalanced panel and a parametric and linear statistical model is discussed in Pitt (2021). We will compare the difference in two-way contingency tables analysis versus fixed effect negative binomial estimation in the section “Two-way contingency tables compared to FENB estimation”.

The method used by Amiry and Maguire (2021) in their ‘narrative review’ of previously published Covid-19 studies was a secondary and speculative compilation with anecdotal evidence and news media reports with Google Scholar being the primary source.

The Covid-19 study by Azbel et al. (2021) used what they called a ‘retrospective cohort study’ that involved a non-parametric test called the Mann–Whitney *U* test to validate their model.

The statistical analysis by Xie et al. (2021) involved what was described as ‘change point detection with binary segmentation’ and that appears to be some sort of cluster analysis.

The common statistical flaws in many of the studies mentioned above were that they were mostly non-parametric in nature, important variables such as ambulance types were omitted and most failed to account for statistical issues such as over-dispersion and fixed effects. The underlying special case of the Poison distribution such as the negative binomial in EMS call patterns was not considered in some studies.

Furthermore, Ioannidis et al. (2022) have clearly documented that the early forecasting efforts there were predicting exponential Covid-19 cases and deaths were failures due in part to ‘poor data input, wrong modeling assumptions, high sensitivity of estimates, lack of incorporation of epidemiological features, poor past evidence on effects of available interventions, lack of transparency, errors, lack of determinacy, consideration of only one or a few dimensions of the problem at hand, lack of expertise in crucial disciplines, group-think and bandwagon effects, and selective reporting.’

## Statement of the problem

### Study motivation

To overcome some of the statistical challenges facing fire and EMS analysis—as discussed in Henderson (2009), Ingolfsson (2013), and Zaric (2013)—and following up on the recommendations of Ioannidis et al. (2022) to improve data modeling in the Covid-19 pandemic era, we provide a more sophisticated econometric (parametric) analysis.

We use extensions to Poisson distribution theory in which comprehensive public policy decisions can be decided based on *inferences* made from clustered standard errors, *p*-values, and confidence intervals, associated with the estimated coefficients.

Therefore, the accuracy, consistency, and reliability of estimated parameters—using Wald, likelihood ratio (LR), and Lagrange multiplier (LM), AIC, and BIC tests found in econometric literature—and model fit can be verified and reproduced in a non-arbitrary way.

From a public policy perspective, our investigation was illuminating because we compared and contrasted the before and after the state of FDNY’s system-wide performance in order to improve city health services to vulnerable populations and other residents in a future crisis.

### Objectives

The overall aim of this study is to fix the underlying statistical problems of some of the previous studies cited earlier on EMS call patterns before and after the Covid-19 pandemic that were one-dimensional or non-parametric in some cases. The first objective of this paper is to determine the relative contribution of medical emergencies and other factors in predicting ambulance travel or response times using a *fixed effects negative binomial* (FENB) model as discussed in Berge (2018) and Cameron and Miller (2015).

Our model uses key FDNY EMS data that includes *City Performance Indicators* such as daily *emergency call types*, clinical *priority rank*, *ambulance types*, (Dual/ALS/BLS), and EMT/paramedic crew data that were often omitted from previous studies. Second, we address the non-parametric limitations of other Covid studies that may include two-way contingency tables.

## Method

### Data sources and study variables

In designing our purely numerical study that did not involve human subjects, we accessed data from two primary sources. The first source of the raw data before aggregation is a subset of the FDNY’s *EMS Incident Dispatch Data* Specifically, we use the following FDNY’s EMS variables: Borough, Cad_Incident_Id, Incident_Datetime, Final_Call_Type, Incident_Response_Seconds_Qy, Incident_Travel_Tm_Seconds_Qy, and Incident_Disposition_Code.^1^

The second source is the *NYC 911 Ambulance Call Types, Priority and Response* report that is available through the *New York State Volunteer Ambulance & Rescue Association, Inc*. and it is assumed to be an accurate representation at the time of writing. Specifically, we merged the FDNY’s EMS Incident Dispatch Data with the NYC 911 Ambulance Call Types, Priority, and Response report. Table 1 provides an overview of the variables used in developing our model.

**Table 1:** Data Description and Sources.

Variable	Description	Source	Unit
Borough	Five boroughs of NYC	EMS Incident Dispatch Data	Factor
Cad_Incident_Id	Daily sequenced number of each 911 incident	EMS Incident Dispatch Data	Count
Incident_Datetime	Date and time of each Cad Incident	EMS Incident Dispatch Data	Date
Final_Call_Type	Call type at the time incident closes	EMS Incident Dispatch Data	Factor
Incident_Travel_Tm_Seconds_Qy	The time elapsed in seconds between the First_Assignment_Datetime and the First_On_Scene_Datetime.	EMS Incident Dispatch Data	Count
Incident_Disposition_Code	A code indicating the final outcome of the incident.	EMS Incident Dispatch Data	Factor
Ambulance Response By Priority & Call Type	Ambulance response type by life/non-life threatening emergency Priority	NYC 911 Ambulance Call Types, Priority, and Response	Factor

Sources: https://data.cityofnewyork.us/Public-Safety/EMS-Incident-Dispatch-Data/76xm-jjuj.

https://www.nysvara.org/

### Study setting

In Fig. 2, the timed and distinct stages and the critical communications link in the EMS dispatch and response process flow such as response time categories, time intervals, and *operational* (as distinct from clinical) benchmarks or performance measures are illustrated. There is a relationship between the number of calls or counts and duration (the time that has elapsed between calls) with the FDNY data. In between each call or duration, ambulances and other certified first responders (CFRs) are dispatched to each event; and the critical timing of such events is recorded.

Two important operational benchmarks of an EMS system are the *total response time* (the time interval between *alarm transfer* and *first-on-scene* time) and *unit response* time (the time between when the EMS units are dispatched and the EMS units arrival on the scene). *Travel or response time* as illustrated—by the shaded area in the process flow figure—measures the proportion of emergency 911 calls that can be responded to within a predefined benchmark by EMS agencies.

Response or travel time is often used as an important operational or performance measure for a variety of reasons, including resource allocation decisions such as whether to open or close call centers, types of ambulance crew and equipment deployed, and staffing levels at EMS call centers. The focus of this study is on the *travel time* portion of unit response time in which the ideal NFPA benchmark is 4 min or less in responding to an emergency will be used as the dependent variable used in this study. Paramedic and EMT response time to EMS incidents is often critical because the faster that first responders arrive on the scene, the greater the likelihood of preventing a fatality.

The notification methods (including miscommunications among all parties), staff training, dispatcher experience, facility layout, ambulances in a ready-state, ambulances that are off-service due to vandalism, EMTs and paramedics availability, tasks at the time of alarm, etc.), weather conditions, traffic congestion, and road construction are all factors that can increase or decrease mobilization, and that in turn can affect *turnout* times in a dispatch center.

Prior to responding to an emergency, Moore-Merrell (2019) suggests that there are three basic internal components of response time that may affect high or low performance: *availability*—the degree to which the resources are ready and available to respond; *capability*—the abilities of deployed resources to manage an incident and *operational effectiveness*, a product of availability and capability.

The faster emergency medical crews arrive on the scene, the more likely the number of injuries, deaths, property damage, and other losses can be minimized. Therefore, a lower time interval in an EMS response to emergencies is associated with the efficient allocation of call center staffing, EMTs, paramedics, and ambulances. On the other hand, higher EMS travel time may be linked to internal financial, crew, training, or equipment inefficiencies and the drastic need for improvement (Blackwell and Kaufman, 2002; Blanchard et al., 2012; Neil, 2009; Wilde, 2012).

### Sample size

Table 2 is a high-level overview of the initial 611,276 observations used in our sample before final aggregation in which we compared the year-over-year changes in emergency calls to the FDNY for the selected periods of March 20, 2019–June 13, 2019 and March 20, 2020–June 13, 2020, the lockdown period in New York City. The table shows the daily call volume in each respective time period, March 20–June 13 for the years 2019 and 2020 for a total of 611,276 that were handled by FDNY dispatchers.

**Table 2:** Selected Y/Y FDNY daily call volume, (%) shares, change, and (%) change (March 20–June 13) period.

Day	2019 counts	2020 counts	Total counts	2019 shares (%)	2020 shares (%)	Total share (%)	2019–2020 change	2019–2020 change (%)
Thu	48,748	39,637	88,385	55.15	44.85	14.46	−9111	−18.69
Wed	49,366	40,690	90,056	54.82	45.18	14.73	−8676	−17.57
Tue	44,997	40,962	85,959	52.35	47.65	14.06	−4035	−8.97
Mon	45,681	41,694	87,375	52.28	47.72	14.29	−3987	−8.73
Sun	41,407	39,269	80,676	51.33	48.67	13.20	−2138	−5.16
Fri	46,173	45,765	91,938	50.22	49.78	15.04	−408	−0.88
Sat	43,379	43,508	86,887	49.93	50.07	14.21	129	0.30
Total	319,751	291,525	611,276	52.31	47.69	100.00	−28,226	−8.83

Call types (EVAC, EVENT, Standby, Transfer, and NA); disposition codes (86, 87, 90, and NA); priority codes (8 and 9) and Incident_Travel_TM_Seconds_QY with a missing timestamp are all excluded.

### Fixed effects negative binomial model (FENB)

One key assumption of the Poisson distribution is the equality of mean and variance which means that there is a constant arrival rate for each emergency call arriving at an FDNY call center, sometimes referred to as the equi-dispersion assumption. When the equi-dispersion assumption is violated, over-dispersion is said to be likely present and this adds ‘extra’ heterogeneity to the data. In practice, the flexible negative binomial variant is often the Poisson model that is generalized as a gamma mixture distribution with fixed and random effects as discussed in Cameron and Trivedi (2014) and Wooldridge (2010).

There have been recent advances in improving the efficient estimation of NB models with *multiple* fixed effects and using maximum likelihood (ML) methods based on the works of Berge (2018) and Cameron and Miller (2015). Thus the optimization procedure is done only on the coefficients of interest, while the fixed effects are dealt with separately in the concentrated likelihood in order to obtain the asymptotic standard errors of the coefficients of interest.

Using Berge (2018)’s method and R Core Team (2021), we provide a practical approach to modeling NYC 911 emergency calls count data using an NB model with multiple fixed effects (FENB) when over-dispersion. is suspected and includes key operational aspects of FDNY’s emergency calls. Equation (1) illustrates the negative binomial model using maximum-likelihood estimation with multiple fixed effects model used in our estimation:

1 $${y}_{ijt}={\beta }_{0}+{\beta }_{t}({\rm {Year}})+{\beta }_{i}({\rm {Day}})+{\beta }_{j}({\rm {Boro}})+\left({\beta }^{\prime}{x}_{ijt},\theta \right),$$

where *y*_*ijt*_ is the average travel time (ATT) that is represented by the shaded area in Fig. 2; *t* = 2019 and 2020; *i* = Monday, Tuesday, Wednesday, Thursday, Friday, Saturday, and Sunday; and *j* = Bronx, Brooklyn, Manhattan, Queens, and Staten Island. year, day and boro are three sets of fixed effects and *x*_*ijt*_ is a data frame of FDNY’s call volume data including aggregated call types, clinical priority, ambulance response (DUAL, ALS, BLS) for the periods March 20, 2019–June 13, 2019 and March 20, 2020–June 13, 2020, respectively. *β*_0_ is an intercept term that is estimated separately and the other $\beta ^{\prime} {\rm {s}}$ are the estimated elasticities of interest with the marginal contributions of each variable. Finally, *θ* is an estimated over-dispersion parameter for the entire model.

It is worth noting according to the FDNY guidelines that both initial_call_type and final_call_type are usually the same and “[do] not reflect the actual condition of the patient. It is a determination based on information obtained from the caller for the purpose of defining severity and resource allocation. The call type does not change based on the findings of the on-scene ambulance crew.”

## Results

Table 3 displays the summary from our FENB estimation of the effects of call volume year, day of the week, assigned call types, priority, ambulance type, and borough on average travel or response time. Several factor levels within the category variable were aggregated as follows. “Choking” call type was added as a categorical level under Cardiovascular. “Caller NotSpecific”, “Fire Police” and “Unknown Cond” were added as levels under *Other* to create the *non-medical* related category. The reference or base level for each categorical variable is Year = “2019”; Day = “Fri”; Category = “Other”; RespPrior = “BLS7” and Boro = "Manhattan”. The reference level for Call types in 2020 *only* is “CallTypeNo2019”, which is used as an ‘offset’ to account for a substantial number of call types that appeared only in the Year 2020 and there was no matching data for 2019. The column labeled “Estimate” contains the log(mean) or estimated elasticities of the independent variables in the model with different sets of uncorrected and corrected FE standard errors. The column labeled “Odds Ratio”, sometimes called an incidence rate ratio (IRR), is the estimated elasticities that have been exponentiated to simplify the results explanation. For example, log(Incidents) has an estimated value of −0.0300 which gives us an odds ratio of exp(−0.0300) = 0.9705 and an odds ratio (%) of 2.9544%.

**Table 3:** Summary estimated NB model coefficients with uncorrected and corrected FE standard errors.

Uncorrected FE standard errors							Corrected clustered standard errors			
Variable	Estimate	Odds ratio exp (*β*)	Odds ratio (%)	Std. error no FE	Statistic	*p*-value	Std. error clustered FE	Statistic	*p*-value	Likely effect *p*-value < 0.05
Intercept	6.4829	653.8747	–	0.0178	363.6177	0.0000	0.2400	27.2300	0.0000	
Emergency Incidents (Call Volume)										
log(Incidents)	−0.0300	0.9705	2.95	0.0023	−13.2761	0.0000	0.0100	−4.6200	0.0000	
Year (2019 vs. 2020)										
Y2020	0.0134	1.0135	−1.35	0.0064	2.0841	0.0371	0.0700	0.2000	0.8400	No effect
*Day of the week*										
Wed	−0.0716	0.9309	6.91	0.0113	−6.3106	0.0000	0.0300	−2.0600	0.0400	
Sun	−0.0517	0.9496	5.04	0.0114	−4.5407	0.0000	0.0900	−0.5800	0.5600	No effect
Sat	−0.0302	0.9702	2.98	0.0113	−2.6828	0.0073	0.0800	−0.3600	0.7200	No effect
Thu	−0.0243	0.9760	2.40	0.0114	−2.1366	0.0326	0.0500	−0.5000	0.6200	No effect
Tue	−0.0030	0.9970	0.30	0.0113	−0.2685	0.7883	0.0800	−0.0400	0.9700	No effect
Mon	0.0949	1.0995	−9.95	0.0113	8.3823	0.0000	0.0700	1.3900	0.1600	No effect
Category (grouped call types)										
Infection	0.5187	1.6798	−67.98	0.0156	33.1554	0.0000	0.1600	3.3200	0.0000	
Abdominal pain	0.4147	1.5139	−51.39	0.0250	16.5709	0.0000	0.1100	3.7900	0.0000	
Cardiovascular	0.3487	1.4172	−41.72	0.0161	21.6895	0.0000	0.0800	4.5000	0.0000	
Neurological	0.2795	1.3224	−32.24	0.0213	13.1229	0.0000	0.1100	2.4800	0.0100	
Respiratory	0.2768	1.3189	−31.89	0.0162	17.1236	0.0000	0.1300	2.1700	0.0300	
Psych	0.2694	1.3092	−30.92	0.0173	15.5712	0.0000	0.0600	4.6900	0.0000	
Obstetrics	0.2421	1.2739	−27.39	0.0179	13.5211	0.0000	0.0900	2.8000	0.0100	
Unconscious	0.2404	1.2718	−27.18	0.0240	10.0194	0.0000	0.1200	1.9600	0.0500	
Injury	0.2018	1.2236	−22.36	0.0145	13.9598	0.0000	0.0500	3.7400	0.0000	
Allergy	0.1973	1.2181	−21.81	0.0239	8.2728	0.0000	0.1100	1.7800	0.0700	No effect
Trauma	0.1120	1.1185	−11.85	0.0168	6.6861	0.0000	0.0500	2.4500	0.0100	
Alcohol drugs	0.0926	1.0970	−9.70	0.0193	4.7902	0.0000	0.0500	1.9600	0.0500	
*EMS priority & Ambulance Assigned*										
DUAL1	−0.9575	0.3839	61.61	0.0215	−44.5785	0.0000	0.0600	−16.9600	0.0000	
DUAL2	−0.7942	0.4519	54.81	0.0707	−11.2327	0.0000	0.1200	−6.7700	0.0000	
BLS3	−0.7052	0.4940	50.60	0.0168	−41.9172	0.0000	0.0300	−26.9400	0.0000	
BLS2	−0.6471	0.5236	47.64	0.0167	−38.6532	0.0000	0.0400	−17.5200	0.0000	
ALS2	−0.5924	0.5530	44.70	0.0191	−30.9741	0.0000	0.0500	−10.9600	0.0000	
ALS3	−0.5022	0.6052	39.48	0.0140	−35.8724	0.0000	0.0800	−6.6600	0.0000	
BLS4	−0.1984	0.8201	17.99	0.0143	−13.8776	0.0000	0.0400	−5.0800	0.0000	
BLS5	−0.1157	0.8907	10.93	0.0163	−7.1102	0.0000	0.0200	−4.8500	0.0000	
BLS6	−0.0566	0.9450	5.50	0.0165	−3.4248	0.0006	0.0700	−0.8600	0.3900	No effect
*NYC Borough*										
Staten Island	−0.2113	0.8095	19.05	0.0110	−19.1515	0.0000	0.0700	-3.0200	0.0000	
Bronx	0.2770	1.3192	−31.92	0.0092	29.9589	0.0000	0.1200	2.2700	0.0200	
Brooklyn	0.1214	1.1290	−12.90	0.0092	13.1993	0.0000	0.1300	0.9600	0.3400	No effect
Queens	0.0352	1.0358	−3.58	0.0094	3.7416	0.0002	0.1700	0.2100	0.8300	No effect
*Offset for missing Call Types*										
CallTypeYes2020	0.0374	1.0381	−3.81%	0.0113	3.3141	0.0009	0.0600	0.6700	0.5000	No effect
*Dispersion parameter*										
*θ*	3.2171	24.9549	–	0.0237	135.9965	0.0000	0.7700	4.2000	0.0000	

nobs = 33,949.00, AIC = 483,228.08, BIC = 483,523.22, logLik = −241,579.04, pseudo.r.squared = 0.0239 and Squared Cor. = 0.1039780, Wald statistic = 916.1, *p*-value < 0.0000 on 35 and 33,914 DoF.

We can interpret the estimated coefficient on the log(Incidents) as follows. The estimated odds of average travel time is equal to 2.9544% or close to 3% for each additional emergency call, given that all the other variables are held constant. The other elasticities are interpreted in a similar manner. Looking at the Year 2020, we see that the unit change in average travel or response time declined by −1.3526% when compared to the pre-lockdown year in 2019 and the FE standard errors were uncorrected. However, when the FE standard errors are corrected Y2020 (*p*-value = 0.8400), statistically speaking, there appears to be no significance between the two years. Wednesday is the only day that is significant when compared to the omitted day of Friday and it is the day with the largest increase in response time of 6.91%.

When combined, life-threatening medical emergencies categorical levels such as infection abdominal pain, cardiovascular, neurological, respiratory, psych obstetrics, unconscious, injury, trauma, and alcohol drugs, travel time declined by 27.24% when allergy is excluded and the comparison is made to the base or omitted level of *Other* that contained non-life threatening emergencies or police and fire-related emergencies.

In the year-over-year comparison, clinical priority 6 and BLS ambulance (BLS6) had no statistical significance when compared to the omitted level of BLS7. However, travel times increased with all the other levels with DUAL 1 (61.6140%) and DUAL 2 (54.8075% responses that required both ALS and BLS equipment lead the year-over-year increased average travel times.

Across the five NYC boroughs, Brooklyn and Queens appear to have no statistical significance when they are compared to the omitted level of Manhattan. Staten Island is the only borough that saw travel time increase by 19.0473%, while travel time declined in the Bronx by 31.92% The offset variable that was used to account for the substantial number of call types that appeared only in the Year 2020 and there was no matching data for 2019 is not particularly significant when the error terms are corrected for fixed effects.

In a timed EMS process, unobserved heterogeneity can be introduced into the system and captured by factors—such as gender, age, race, health condition, health insurance coverage, population size, location (private residence, business, nursing home, or long-term care facility), income, education, employment and unemployment, uneven emergency call patterns during the year, month or day, the priority assigned to the call, the ambulance or CFR crews assigned to the call, seasonal factors, geological or weather conditions, unpredictable events such as the Covid-19 Epidemic and other unknown factors—and they tend to increase over-dispersion within the EMS call volume process.

The estimated over-dispersion parameter (*θ*)—that pertains to the whole model as opposed to non-parametric pairwise estimates—is shown in the table and it is significant, in which case we can conservatively assume there is substantial over-dispersion.

### Corrected clustered standard errors

In Table 3’s presentation, the model’s estimated parameters are shown separately with two sets of standard error calculations. In the column labeled ‘Std. error no FE’ is the first set of estimated standard errors with their corresponding Wald test statistic and *p*-value *without* a correction for fixed effects clustered standard errors in the presence of over-dispersion.

When such an estimation is conducted and the panel (multi-dimensional data that is collected over time) is “partitioned into different clusters, treating each observation as independent from the others leads to [underestimating] the variance of the coefficient.” In other words, you get standard errors that are too small, narrow confidence intervals, inflated *t*-statistics, and misleadingly small *p*-values (Berge, 2018; Cameron and Miller, 2015).

To correct for fixed effects, the model is re-estimated with clustered standard errors that are shown as the second set of standard errors in the column labeled ‘Std. error clustered FE’. This adjustment produces accurate standard errors which in turn is a critical component when making statistical inferences. It is important to point out that the estimated coefficients remain the same with or without the corrected errors. However, the expected values of the corrected standard errors are now much higher than in the uncorrected case when the observations were considered independent.

## Discussion

In this study, we used an aggregated sample of 33,949 FDNY’s EMS calls for the pre-lockdown period of March 20, 2019–June 13, 2019, and the and post lockdown periods of March 20, 2020–June 13, 2020, with the goal of determining the system-wide effects of FDNY’s EMS key City Performance Indicators data such as daily call volume, year, day of the week, call types, clinical priority rank, ambulance types, NYC boroughs, an offset parameter and a dispersion parameter in predicting ambulance crew travel or response time using a fixed effects negative binomial regression model. Ambulance types (Dual/ALS/BLS) were often omitted from previous studies and this may be one of the recent studies to include those variables.

Specifically, in estimating a fixed effect negative binomial (FENB) model, we used recent econometric methods that efficiently estimate maximum likelihood (ML) models with any number of fixed effects and easily obtained clustered standard errors with an algorithm that is based solely on the concentrated likelihood (Berge, 2018; Cameron and Miller, 2015). This method could be an improvement in quantifying uncertainty often associated with other models.

The modeling of EMS call center data was considered problematic, rudimentary, and quite often mis-specified, depending on which statistical method was used. More often some of these studies used a mixture of non-parametric and parametric methods that did not capture the key underlying properties of the associated statistical distributions and the complex processes involved in a modern EMS system (Henderson, 2009; Matteson et al., 2011). During the two yearly periods, some form of pre-hospital medical aid was provided to patients who remained on the scene upon EMS arrival. There were 319,751 calls during 2019 and 291,525 calls in 2020 for a total of 611,276, after call types (EVAC, EVENT, Standby, Transfer, and NA); disposition codes (86, 87, 90, and NA), Priority codes (8 and 9) and calls with an Incident_Travel_TM_Seconds_QY timestamp missing were all excluded. Surprisingly in 2020, the total number of emergency calls declined by 28,226 when compared to 2019, or a year-over-year decrease of 8.83%.

In the column labeled Total Share (%), we see that Fridays with a count of 91,938 calls 15.04 (%) followed by Wednesdays with a count of 90,056 (14.73)% calls and Thursdays 88,385 (14.46%) were generally the busiest days of the weeks for emergency calls into the FDNY with a cumulative total of 44.23%. Every weekday—except for a minor increase on Saturday—saw declines in call volume, with major declines appearing to have occurred on Thursdays (18.69%) and Wednesdays (17.57%). A drill-down of the data in Table 2 is reported in Supplementary Information without commentary for the reader’s perusal.

In creating the cross-section panel for model estimation, it was necessary to aggregate the raw sample of 611,276 observations by call or incident type because ambulance crews could have been dispatched to the same type of call on multiple occasions on the same day in different boroughs. Following data aggregation, the raw data was reduced to 33,949 observations that were used for model estimation.

The daily call volume received by the FDNY’s emergency call centers is often modeled as counts, given the nature of its discreteness, nonlinear and non-negative values, and such calls are said to follow a negative binomial distribution when the outcome variables may be over-dispersed. This is the case where the conditional mean and conditional variance of 911 emergency incidents by medical and non-medical call type per second are not equal. The negative binomial is considered a special case of the Poisson distribution with an extra parameter added to account for over-dispersion. It is often difficult to tell if over-dispersion is significant in a model without estimation.

**Fig. 1:**
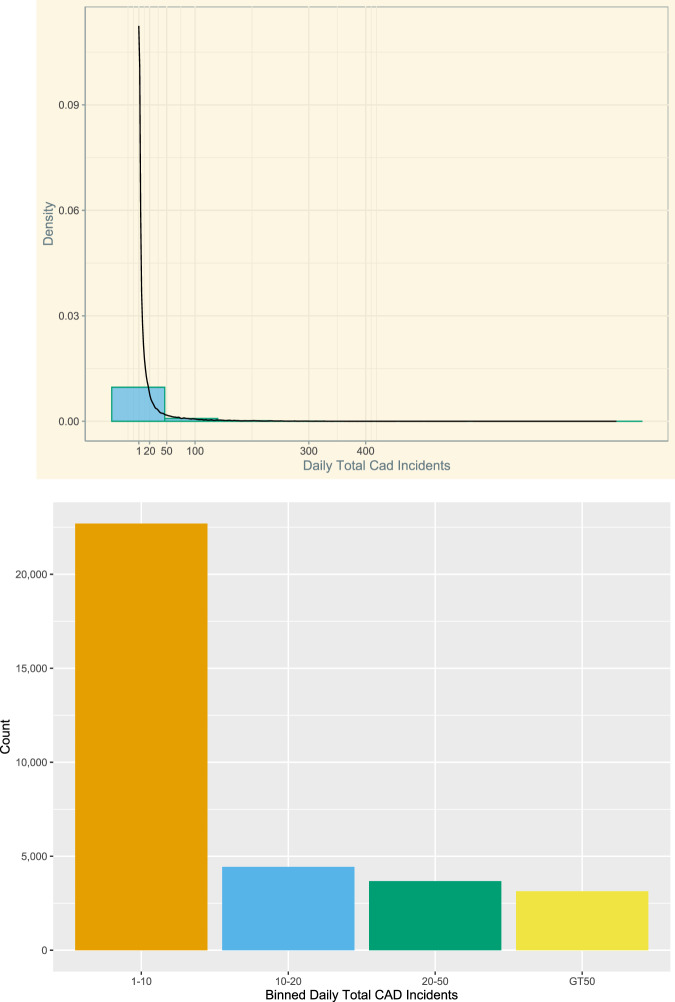
Frequency distributions of FDNY’s Daily EMS Incidents (Call Volume) Yrs 2005–2020. **a** Histogram EMS incidents. **b** Binned EMS incidents.

A high-level overview of Emergency CAD Incidents or call volume from our initial data set is shown in Fig. 1, for the years 2005–2020. Plot (a) is a histogram of count frequencies of daily call volume and with a density, curve added it shows the data is not normally distributed. In other words, the figure depicts a negative binomial distribution that is a special case of the Poisson distribution. Plot (b) histogram (when the number of incoming calls is binned into four-factor levels—“1–10”, “10–20”, “20–50” and “GT50”—for illustrating in better detail the clustering or clumping of large counts of data in the distribution) show that on a daily basis FDNY’s call centers received between 1 and 10 calls *per* emergency incident call type or thousands of calls each month for a city with 8.4 million residents. The “clumping” around 1–10 daily calls per call type suggests that the variance is likely larger than the mean and this could result in over-dispersion.

In Moller et al. (2015) study of EMS call patterns, they used a negative binomial model that included a clinical priority level but differed from our study with the exclusion of ambulance types and other factors. Interestingly, they made two types of adjustments to test for over-dispersion. First, they used a pairwise comparison of categories of ‘significant variables’ with a Pearson dispersion parameter to assess the goodness-of-fit of their model and found the estimated parameter (1.92) to be ‘adequate.’ What statistical criteria were used to determine that the parameter was inadequate is not discussed and the author is not sure what inadequate means in a statistical sense.

Having found that their over-dispersion parameter was inadequate, they then performed a ‘sensitivity analysis, modeling the data with a negative binomial distribution, resulting in a dispersion parameter of 1.16.’ It was hard to evaluate this study for comparative purposes because no standard errors, test statistics, or *p*-values were reported in a format similar to ours in Table 3. Furthermore, there is no mention of whether the standard errors were corrected or uncorrected or fixed effects were considered as suggested by the model discussed in this paper. It would have been useful to have a table with a comparison of the Pearson and negative binomial test results to increase the reader’s confidence in their final results selection criteria.

**Fig. 2:**
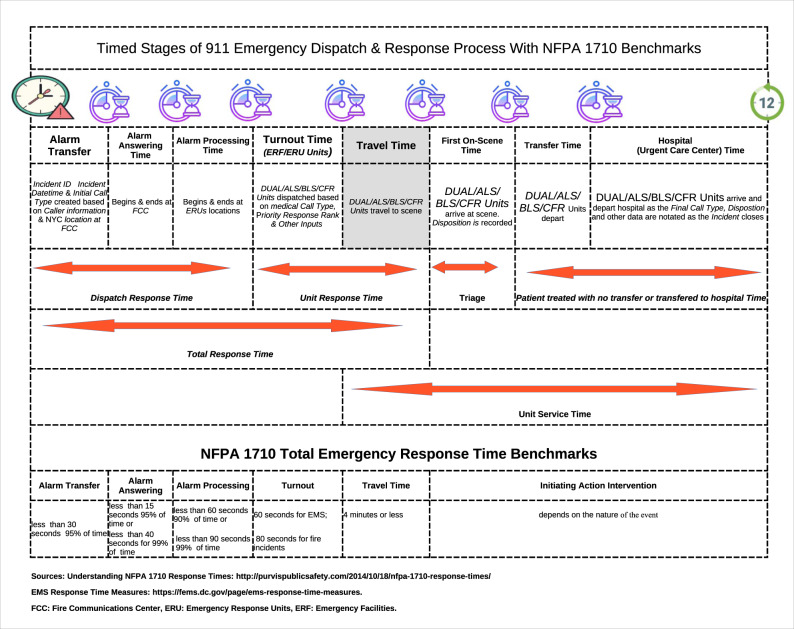
Timed stages of EMS dispatch and response process with benchmarks. Is a pictograph of an emergency call process flow.

One important distinction between this paper and many of the non-parametric studies is that over-dispersion was justified by the pattern first observed in Fig. 2. As shown in Table 3, over-dispersion was significant (*θ* = 3.2171, std. err. clustered FE = 0.7770, Statistic = 4.2000 and *p*-value = 0.0000) in the model with corrected clustered standard errors. More importantly, our dispersion parameter was estimated using the full information from the entire sample and it is only in post-estimation analysis that over-dispersion can really be determined to be significant or not.

In addition, when faced with the decision criteria for model selection involved in nested designs, the summary statistics criteria shown in Table 4 are clearly well-defined for decision-making.

**Table 4:** Decision criteria and summary statistics of full and reduced models with correct FE standard errors.

Statistics	Full model	Reduced model
pseudo.r.squared	0.0255	0.02544
nobs	16,865	16,865
AIC	237,861	237,896
BIC	238,047	238,074
logLik	−118,907	−118,925

### Two-way contingency tables compared to FENB estimation

For comparative purposes to two-way contingency tables (non-parametric analysis) of categorical variables and the ratios of various cell’s proportions, average travel time, the dependent or outcome variable is actually a count, which is the average travel time per call type on a given day and borough.

The column labeled ‘Ratio exp(*β*)’ in Table 3 from the FENB estimation is the incidence rate ratio (IRR). So that when we exponentiate the following ratio:

$${\rm {log(TravelTime| Category)}}\,=\frac{{\rm {log(TravelTime| Cardiovascular)}}}{{\rm {log(TravelTime| Other)}}},$$

we get the odds ratio or incident rate ratio (IRR) shown as Cardiovascular exp(*β*) = 1.4172 and explained in the “Results” section. This is the adjusted ratio of the average travel time for cardiovascular calls when compared to the average travel time for call types that were aggregated into levels labeled Other and served as the omitted, base, or reference level when all the other variables are held constant.

The relative size indicates the relative strength of each variable’s effect rather than their marginal impact in contrast to linear models (Cameron and Trivedi, 1986). The difference between our analysis and the presentation in Prezant et al. (2020) is that we have utilized the underlying statistical properties of the Poisson distribution extension in a multivariate parametric approach by analyzing the simultaneous effects of several explanatory variables rather than just a two-way contingency table with limited information. See Agresti (2007, Chapter 2, pp. 21–64) for an analysis of the association between categorical variables and the computation of relative risk and odds ratios.

In this paper, we have fixed two potential problems with estimating EMS call center data. First, we used actual real-world EMS data from the FDNY to model travel or response time using the underlying statistical distribution, a negative binomial as a special case of the Poisson distribution.

Second, the rather opaque monthly FDNY’s Citywide Performance Indicators report^2^ can be vastly improved and communicated to a wider audience with the addition of other variables—such as day of the week, call types grouped into medical categories, priority, ambulance type, patient demographic data (race, gender, and age) along with various travel times—in a more transparent format that would resolve any inaccuracies that would be due to the fact that not all of the Citywide Performance Indicators are readily available in one place at the website. An updated EMS Incident Dispatch Data file would also better reflect current EMS best practices in data standardization that could be used across the country, simplify the munging of EMS data, and would help researchers better understand the complicated data.

## Model aptness

In developing a statistical model, the researcher is often faced with the difficulty of variable selection that makes the best contribution to a model based on using fewer parameters that are significant, particularly when using a nested approach. In a maximum-likelihood framework, evaluation of FENB models and diagnostic testing would include the usual staple in econometrics such as the Wald Test on whether all of the estimated coefficients are equal to zero. Other tests, such as the likelihood ratio (LR), Lagrange multiplier (LM) along with AIC, BIC, and pseudo-*r*^2^ statistic, are more often used when more than one or nested models are compared (Engle, 1984). Table 5 is the Wald tests of joint nullity of individual independent variables and the whole model that was estimated. It is often used as a diagnostic test of the relative importance of certain variables in order to determine whether dropping variables would improve the model fit or make the model more parsimonious. We note that all of the *p-*values are below the 0.05 threshold. This suggests that the estimated coefficients are not all simultaneously equal to zero and all of the variables can be retained in the model. Therefore, there is no practical purpose in fitting an initial model with a reduced number of variables to make the model estimation more parsimonious.

**Table 5:** Wald test statistics of joint nullity by multiple variables.

Variable	Wald *T*-statistic	*p*-value	First DOF	Second DOF
Emergency incidents (call volume)	176.30	0.0000	1.00	33,914
Year (2019 & 2020)	4.34	0.0372	1.00	33,914
Day of the Week	44.40	0.0000	6.00	33,914
Category (grouped call types)	129.10	0.0000	12.00	33,914
EMS priority & ambulance assigned	510.20	0.0000	9.00	33,914
NYC borough	561.50	0.0000	4.00	33,914
Offset for missing call types	10.10	0.0009	1.00	33,914
Joint nullity (all variables)	916.10	0.0000	35.00	33,914

### Residual diagnostics

Further graphical analysis is sometimes conducted to assess the appropriateness of model fit following estimation, even though it is rarely reported. A visual residual analysis would include a check for skewness, outliers, and other influential observations that would indicate specification errors in the chosen distribution. There are several options when it comes to plotting residuals following a negative binomial estimation and a Deviance residual plot is among the various classes that would also include Pearson and Quantile residuals. We used the Deviance residual class because it tends to reveal patterns that may not be apparent in other classes as discussed in Dunn and Smyth (2018).

**Fig. 3:**
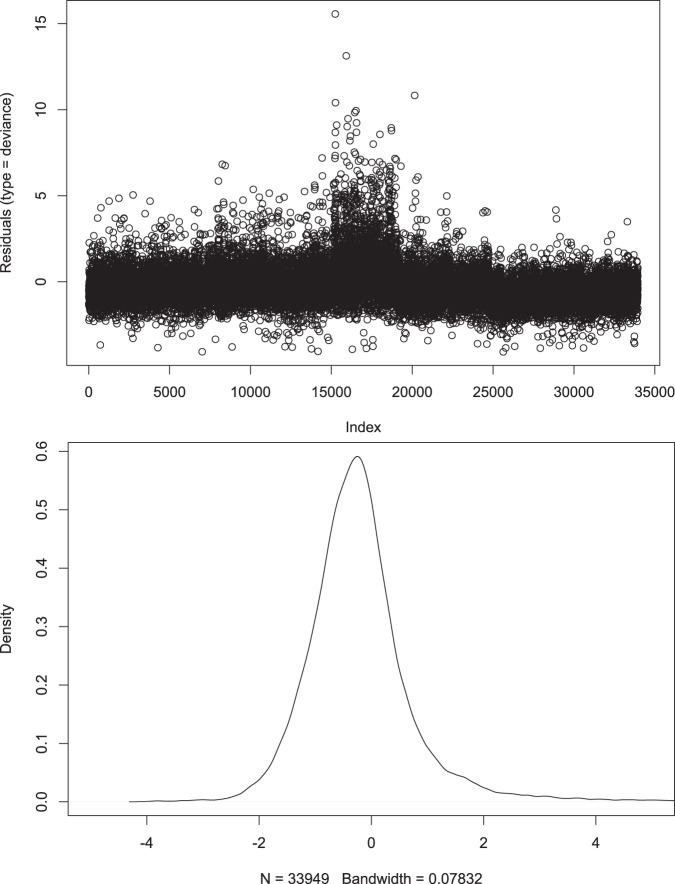
Deviance and density residual plots. **a** Deviance residual plot. **b** Deviance residual plot with a density curve added.

Figure 3a shows the Deviance residual plot for our data. It shows the negative binomial residuals roughly distributed around the zero line and there is no discernible pattern that would indicate any of the NB assumptions were violated. Figure 3b is a smoother depiction of Fig. 3a in which a density kernel has been added. In this plot, it can be clearly seen that the residuals are close in approximation to a normal distribution and this is not surprising given the large sample size that was used.

When the results from the estimated *θ* dispersion parameter, the Wald tests, and Deviance residual graphical analysis are combined we can safely assume that the negative binomial model is more appropriate in this case rather than the Poisson model when the presence of over-dispersion is detected.

### Model reduction

The real interest in developing statistical models is quantifying which variables are relatively important in predicting a likely outcome. Day of the week would be likely important in predicting the demand for pre-hospital care at least from a call center (training and staffing) administrative perspective. Is there a demand for pre-hospital care on certain days of the week or times during warmer weather versus colder days or on the day before or after major holidays? For example, in our model when we controlled for each day of the week, Wednesday (WED) was the only day that was significant in the reduced model with the corrected standard errors. This raised an important specification question as to whether this was caused by a possible correlation among the independent variables. One method to assess whether this is the case is to estimate the reduced model with and without the *wed* level in the *day* variable and this analysis is shown in Table 6. In comparing the two models in Table 6, the AIC, BIC, and loglik for the model that includes the day = wed variable are all slightly smaller than the model without and that would lead to the conclusion that the model with the day variable is a slightly better fit.

**Table 6:** Summary Estimated NB Reduced Model Coefficients With Corrected FE Standard Errors.

Variable	Estimate	Std. error	Statistic	*p*-value	Estimate	Std. error	Statistic	*p*-value
Intercept	6.4178	0.0405	158.4873	0.0000	6.4080	0.0223	287.5963	0.0000
*Emergency Incidents (Call Volume)*								
log(Incidents)	−0.0266	0.0050	−5.3596	0.0000	−0.0267	0.0032	−8.3793	0.0000
*Day of the Week*								
Wed	−0.0738	0.0361	−2.0421	0.0411	–	–	–	–
*Category (grouped call types)*								
Infection	0.6201	0.1636	3.7911	0.0002	0.6196	0.0276	22.4864	0.0000
Abdominal Pain	0.5232	0.1754	2.9826	0.0029	0.5243	0.0331	15.8479	0.0000
Cardiovascular	0.4143	0.0687	6.0268	0.0000	0.4133	0.0218	18.9945	0.0000
Neurological	0.3932	0.1009	3.8952	0.0001	0.3932	0.0284	13.8313	0.0000
Respiratory	0.3710	0.0935	3.9665	0.0001	0.3712	0.0215	17.2785	0.0000
Psych	0.3308	0.0527	6.2792	0.0000	0.3298	0.0237	13.9101	0.0000
Obstetrics	0.3414	0.1160	2.9426	0.0033	0.3421	0.0242	14.1099	0.0000
Unconscious	0.3534	0.0757	4.6700	0.0000	0.3547	0.0324	10.9623	0.0000
Injury	0.2802	0.0726	3.8607	0.0001	0.2799	0.0203	13.8148	0.0000
Trauma	0.1701	0.0241	7.0447	0.0000	0.1700	0.0232	7.3280	0.0000
Alcohol Drugs	0.1753	0.0592	2.9598	0.0031	0.1748	0.0250	6.9943	0.0000
*EMS priority & ambulance assigned*								
DUAL1	−0.9392	0.1167	−8.0453	0.0000	−0.9384	0.0280	−33.5387	0.0000
DUAL2	−0.8757	0.1988	−4.4056	0.0000	−0.8796	0.0884	−9.9531	0.0000
BLS3	−0.7051	0.0593	−11.8831	0.0000	−0.7055	0.0224	−31.5105	0.0000
BLS2	−0.6489	0.0661	−9.8167	0.0000	−0.6489	0.0221	−29.3547	0.0000
ALS2	−0.6363	0.0597	−10.6499	0.0000	−0.6380	0.0261	−24.4580	0.0000
ALS3	−0.5116	0.0476	−10.7390	0.0000	−0.5119	0.0182	−28.1269	0.0000
BLS4	−0.1749	0.0386	−4.5332	0.0000	−0.1753	0.0191	−9.1763	0.0000
BLS5	−0.1275	0.0310	−4.1182	0.0000	−0.1275	0.0218	−5.8580	0.0000
*NYC Borough*								
Staten Island	−0.1924	0.0034	−56.1357	0.0000	−0.1926	0.0115	−16.8099	0.0000
Bronx	0.2519	0.0049	51.8974	0.0000	0.2531	0.0095	26.6372	0.0000
*Dispersion parameter*								
*θ*	3.4374	0.7343	4.6809	0.0000	3.4305	0.0359	95.4973	0.0000
*Summary statistics*								
pseudo.r.squared	0.0255				0.0254			
nobs	16,865				16,865			
AIC	237,861				237,896			
BIC	238,047				238,074			
logLik	−118,907				−118,925			

## Limitation

This study has several methodological, data availability, and time period limitations, some of which will be addressed in a future article. First, each emergency call is associated with an individual person and as such, there is an associated name, race, gender, age, marital status, a particular location in a zip code, other socioeconomic factors (education, income), and related illness associated with each person or call that are not considered in this model specification.

There was a huge disparity in Covid death rates that differed by gender, age, and race/ethnicity in 2020, according to the Centers for Disease Control and Prevention (CDC). In the United States, provisional nationwide death certificate data for January–December 2020 showed that ‘COVID-19 death rates were highest among males, older adults[≥85], and [minorities]. The highest numbers of overall deaths and COVID-19 deaths occurred during April and December. The mortality ranked order changed when COVID-19 became the third leading underlying cause of death [375,000] in 2020, replacing suicide as one of the top 10 leading causes of death’ (Ahmad et al., 2021; Olson and Wye, 2020). Did patients transported to hospitals by ambulances die of Covid co-morbidities or from it?

Second, during the Covid-19 lockdown period March 20, 2020–June 13, 2020, in New York City, we saw dramatic changes in FDNY call volume data when compared to the same period in 2019 in which certain call types increased dramatically due to increased demand as you would expect in a medical crisis. Where did the increased call volume originate and what percentage of the increased emergency calls were coming from nursing homes, long-term care facilities, and rehabilitation centers for resuscitation compared to other locations? We do not consider such possibilities in this model.

Third, we did not consider whether travel time and mobilization could have been affected by both the availability and workload of EMTs and paramedics, and by the new protocols and contingency plans put in place to protect first responders from unnecessary exposure to sick patients during the crisis.

Fourth, our model likely captured only a subset of the data during the first wave of the Covid-19 epidemic when the highest number of deaths (78,917) were reported for the weeks ending April 11, 2020, and only if those patients received pre-hospital ambulance care by first calling the FDNY 911 call center. The second wave that followed around December 26, 2020, in which 80,656 is said to have died is excluded (Ahmad et al., 2021).

Fifth, there is also a rather mysterious decline in certain other types of emergency calls in which patients hesitated to dial 911 or decided to avoid ambulances and hospitals altogether out of some sort of perceived pandemic fear of contracting Covid on top of their existing illnesses during the lockdown. Did any of these patients seek alternative modes of transportation for medical diagnosis and treatment elsewhere such as ’urgent care’ facilities that we did not consider here?

All of these unknown factors would tend to increase over-dispersion among call volume and a statistical model that accounts for such factors would be more useful in accounting for unexplained variation in the model.

## Conclusions

The model estimated here fairly captured the underlying assumptions of a negative binomial distribution, particularly the measurement of over-dispersion, fixed effects, and the correction for clustered standard errors in analyzing daily EMS call pattern data. In addition, by including both EMS dispatcher-assigned clinical priority rankings and ambulance crew-assigned variables, our method improved on some of the earlier non-parametric studies that were often one-dimensional, rudimentary, misspecified, or failed to capture the complexity of EMS call center data. More importantly, we addressed some of the important statistical measurement failures that were raised by Ioannidis et al. (2022).

The model can be extended to simulate response times that may involve routing non-critical patients to urgent care centers (UCC) or temporary field hospitals instead of over-crowded hospital emergency rooms during a Federal Emergency Management Agency (FEMA) crisis.

## Supplementary information

Supplementary Information

## Data Availability

The data used in this study were obtained from *NYC Open Data*. *NYC Open Data* is a collection of New York City’s operational and performance data organized by city agencies for the purpose of analysis and is freely available for research. The data can be obtained here: https://data.cityofnewyork.us/Public-Safety/EMS-Incident-Dispatch-Data/76xm-jjuj.

## Footnotes

1 The data is available here: https://data.cityofnewyork.us/Public-Safety/EMS-Incident-Dispatch-Data/76xm-jjuj.

2 See https://www1.nnyc.gov/site/fdny/about/resources/data-and-analytics/citywide-statistics.page.
