# Supplementary material for: The system-wide effects of dispatch, response and operational performance on emergency medical services during Covid-19
Source: Humanit Soc Sci Commun. 2022 Nov 18;9(1):412. doi: 10.1057/s41599-022-01405-z (PMC9672593; doi:10.1057/s41599-022-01405-z)
Supplement: Supplementary file 1 — Supplementary Information [file 41599_2022_1405_MOESM1_ESM.pdf]

1 Supplementary Information

Readers might be interested in seeing some of the raw data that were used prior to data aggregated for modeling. We present the data without commentary.

Table 1: **FDNY Call Volume (%) Shares By Year, Day and Grouped Call Type (March 20—June 13)**

Year	Category	Mon (%)	Tue (%)	Wed (%)	Thu (%)	Fri (%)	Sat (%)	Sun (%)	Total (%)	Cum. Share (%)
2019	Injury	19.94	20.12	20.72	20.08	20.57	21.15	20.11	20.38	20.38
2019	Infection	18.63	18.78	18.50	18.26	17.47	17.17	17.85	18.11	38.49
2019	Cardiovascular	14.59	14.39	14.02	14.23	13.96	12.95	13.26	13.93	52.42
2019	Psych	10.07	10.94	10.94	10.88	10.23	9.26	9.53	10.29	62.72
2019	Respiratory	9.20	8.47	8.27	8.58	8.40	8.30	8.92	8.59	71.30
2019	Alcohol Drugs	6.40	6.70	6.79	6.88	7.59	8.77	8.42	7.33	78.63
2019	Unconscious	4.63	4.40	4.61	4.56	5.34	6.02	5.38	4.97	83.60
2019	Caller NotSpecific	4.48	4.52	4.58	4.72	4.75	4.85	4.68	4.65	88.26
2019	Abdominal Pain	3.91	3.94	3.87	3.83	3.69	3.53	3.75	3.79	92.05
2019	Neurological	3.04	2.70	2.72	2.84	2.82	2.78	2.63	2.79	94.84
2019	Trauma	1.79	1.68	1.74	1.78	1.85	2.01	2.17	1.85	96.69
2019	Obstetrics	1.50	1.55	1.52	1.60	1.49	1.44	1.48	1.51	98.21
2019	Allergy	0.82	0.81	0.79	0.90	0.87	0.82	0.80	0.83	99.04
2019	Unknown Cond	0.54	0.54	0.54	0.48	0.53	0.52	0.57	0.53	99.57
2019	Fire Police	0.20	0.23	0.19	0.18	0.21	0.19	0.21	0.20	99.77
2019	Choking	0.16	0.15	0.10	0.10	0.14	0.13	0.16	0.13	99.90
2019	Other	0.11	0.10	0.09	0.10	0.08	0.11	0.09	0.10	100.00
2020	Infection	19.08	18.34	19.35	18.15	18.63	18.30	18.86	18.67	18.67
2020	Cardiovascular	16.78	16.62	15.81	16.41	15.66	15.64	16.70	16.22	34.89
2020	Injury	14.25	14.67	14.36	14.90	15.29	15.89	14.88	14.90	49.79
2020	Respiratory	13.75	12.98	12.59	12.49	12.55	12.19	13.01	12.79	62.58
2020	Psych	10.28	10.66	11.25	11.25	10.82	9.75	10.24	10.60	73.18
2020	Caller NotSpecific	5.75	5.73	5.82	5.85	6.00	6.13	5.46	5.83	79.01
2020	Alcohol Drugs	4.93	5.46	5.53	5.53	5.84	6.35	5.37	5.58	84.60
2020	Unconscious	4.55	4.62	4.66	4.71	4.82	4.96	4.64	4.71	89.31
2020	Abdominal Pain	3.39	3.29	3.24	3.28	3.12	3.09	3.22	3.23	92.54
2020	Neurological	2.29	2.43	2.31	2.29	2.17	2.34	2.32	2.31	94.84
2020	Trauma	1.63	1.82	1.71	1.80	1.76	2.02	1.90	1.81	96.65
2020	Obstetrics	1.17	1.20	1.18	1.24	1.16	1.21	1.25	1.20	97.85
2020	Unknown Cond	1.07	1.19	1.28	1.07	1.12	1.12	1.06	1.13	98.98
2020	Allergy	0.59	0.55	0.51	0.61	0.57	0.56	0.64	0.58	99.55
2020	Fire Police	0.28	0.26	0.18	0.22	0.25	0.25	0.23	0.24	99.80
2020	Choking	0.12	0.10	0.11	0.11	0.13	0.11	0.11	0.11	99.91
2020	Other	0.09	0.09	0.10	0.07	0.10	0.09	0.10	0.09	100.00

Table 2: **FDNY Tiered Priority and Ambulance Response by Day & (%) Shares (March 20–June 13)**

Year	Priority	Ambulance	Mon (%)	Tue (%)	Wed (%)	Thu (%)	Fri (%)	Sat (%)	Sun (%)	Total (%)	Cum. Share (%)
2019	4	BLS	14.12	14.19	14.90	14.29	13.75	14.29	14.46	19.65	19.65
2019	7	BLS	13.70	14.73	14.55	14.22	14.08	14.17	14.55	14.08	33.73
2019	3	ALS	14.09	14.19	14.74	13.98	14.24	14.59	14.19	13.18	46.90
2019	5	BLS	13.66	13.97	14.65	14.81	14.29	14.39	14.23	12.72	59.62
2019	2	ALS	14.13	13.25	15.00	14.54	13.84	14.77	14.48	11.32	70.94
2019	3	BLS	14.19	13.45	14.32	13.39	13.92	15.60	15.13	9.87	80.81
2019	2	BLS	14.26	14.02	13.93	14.51	14.43	15.00	13.85	8.06	88.87
2019	6	BLS	14.23	14.51	14.80	14.99	14.51	13.29	13.67	7.01	95.88
2019	1	DUAL	14.93	13.76	14.09	13.42	14.77	13.76	15.27	3.94	99.82
2019	2	DUAL	14.29	7.14	10.71	14.29	14.29	10.71	28.57	0.18	100.00
Total	—	—	14.06	14.04	14.63	14.28	14.10	14.47	14.43	100.00	—
2020	4	BLS	14.53	14.38	13.70	13.96	14.59	15.18	13.67	17.97	17.97
2020	3	ALS	14.25	14.66	14.06	14.10	14.96	14.40	13.58	14.21	32.18
2020	2	ALS	14.20	14.52	14.04	13.61	14.83	14.87	13.93	13.48	45.66
2020	5	BLS	14.57	14.53	13.38	14.34	15.35	13.89	13.93	11.60	57.26
2020	7	BLS	14.43	14.80	14.15	13.91	14.56	14.15	14.01	11.42	68.68
2020	6	BLS	14.95	14.14	14.19	14.25	14.41	14.25	13.82	9.89	78.57
2020	2	BLS	14.36	14.61	13.32	13.69	14.92	15.41	13.69	8.66	87.23
2020	3	BLS	14.43	14.56	14.03	13.17	15.16	14.96	13.70	8.03	95.26
2020	1	DUAL	15.57	14.52	13.35	13.82	14.52	15.34	12.88	4.54	99.80
2020	2	DUAL	13.16	15.79	13.16	15.79	18.42	15.79	7.89	0.20	100.00
2020	—	—	14.50	14.52	13.84	13.91	14.82	14.68	13.73	100.00	—

DUAL = ALS + BLS,
DUAL, ALS and BLS response can include CFR (engine company) and Rescue units

Table 3: **FDNY Year/Year Increases in Call Volume Ranked By Grouped Call Types, Priority, Ambulance Response and Description (March 20–June 13)**

Category	Call Type	Priority	Ambulance	Description	2019	2020	Change	% Change
Respiratory	RESPIR	4	BLS	RESPIRATORY DISTRESS	3,565	8,268	4,703	131.92%
Unknown Cond	OTHER	6	BLS	UNKNOWN CONDITION	1,688	3,295	1,607	95.20
Cardiovascular	ARREST	1	DUAL	CARDIAC ARREST	5,105	9,775	4,670	91.48
Injury	VENOM	2	BLS	VENOM (SNAKE BITES)	2	3	1	50.00
Fire Police	PD13	7	BLS	POLICE	263	383	120	45.63
Trauma	DROWN	2	DUAL	DROWNING	29	40	11	37.93
Trauma	SHOT	3	BLS	GUN SHOT WOUND	213	268	55	25.82
Other	JUMPUP	7	BLS	JUMPER UP	94	116	22	23.40
Psych	EDPC	7	BLS	PSYCHIATRIC PATIENT	6,453	7,925	1,472	22.81
Caller NotSpecific	UNKNOW	4	BLS	CALLER HAS NO PT MEDICAL INFO	14,875	16,995	2,120	14.25
Trauma	ELECT	3	ALS	ELECTROCUTION	54	60	6	11.11
Obstetrics	OBOUT	3	BLS	BABY OUT OR IMMINENT BIRTH	65	71	6	9.23
Trauma	BURNMA	3	ALS	MAJOR BURNS 18/ADLT/10/CHILD	239	248	9	3.77
Trauma	STAB	3	BLS	STABBING	963	983	20	2.08
Infection	SICKFC	6	BLS	SICK - COUGH & FEVER	0	10,055	10,055	Increase
Respiratory	DIFFFC	2	ALS	DIFF BREATHING – FEVER & COUGH	0	8,429	8,429	Increase
Respiratory	RESPFC	4	BLS	RESP DISTRESS – FEVER & COUGH	0	2,035	2,035	Increase
Cardiovascular	CARDFC	3	ALS	CARDIAC CONDITION-FEVER & COUGH	0	1,857	1,857	Increase
Cardiovascular	ARREFC	1	DUAL	CARD OR RESP ARREST-FEVER/COUGH	0	1,346	1,346	Increase
Abdominal Pain	ABDPFC	5	BLS	ABDOMINAL PAIN-FEVER & COUGH	0	1,040	1,040	Increase
Unconscious	UNCFC	2	ALS	UNC PATIENT - FEVER & COUGH	0	986	986	Increase
Psych	ALTMFC	3	ALS	ALT MENTAL STATUS-FEVER & COUGH	0	985	985	Increase
Respiratory	ASTHFC	2	BLS	ASTHMA ATTACK – FEVER & COUGH	0	758	758	Increase
Infection	PEDFC	6	BLS	SICK PED$<$5 YRS-FEVER & COUGH	0	490	490	Increase
Infection	SICMFC	6	BLS	SICK - COUGH & FEVER	0	482	482	Increase
Trauma	INBLFC	3	ALS	INTERNAL BLEEDING-FEVER & COUGH	0	337	337	Increase
Alcohol Drugs	DRUGFC	4	BLS	HX DRUG OR ALCHL ABUSE-FEV & COU	0	334	334	Increase
Cardiovascular	CVACFC	2	BLS	STROKE CRITICAL – FEVER & COUGH	0	298	298	Increase
Neurological	STATFC	2	ALS	MULT OR PROLONG SEIZUR-FEV & COU	0	249	249	Increase
Cardiovascular	CVAFC	2	BLS	STROKE - FEVER & COUGH	0	177	177	Increase
Allergy	ANAPFC	2	ALS	ANAPHYLACTIC SHOCK-FEVER & COUGH	0	166	166	Increase
Neurological	SEIZFC	3	BLS	SEIZURES - FEVER & COUGH	0	129	129	Increase
Allergy	MEDRFC	5	BLS	REACTION TO MED – FEVER & COUGH	0	32	32	Increase
Choking	CHOKFC	1	DUAL	CHOKING FEVER& COUGH	0	20	20	Increase
Respiratory	RESPRF	4	BLS	RESPIRATORY DISTRESS	0	1	1	Increase
Total	—	—	–	—	33,608	78,636	45,028	133.98%

Table 4: **FDNY Year/Year Decreases in Call Volume Ranked By Grouped Call Types, Priority, Ambulance Response and Description (March 20–June 13)**

Category	Call Type	Priority	Ambulance	Description	2019	2020	Change	(%) Change
Infection	SICPED	4	BLS	SICK PEDIATRIC, <5 YEAR OLD	2,356	664	-1,692	-71.82%
Trauma	HEAT	4	BLS	HEAT EXHAUSTION	42	13	-29	-69.05
Psych	EDPW	7	BLS	PSYCHIATRIC PATIENT	12	5	-7	-58.33
Trauma	AMPMIN	3	BLS	AMPUTATION, FINGERS OR TOES	76	32	-44	-57.89
Injury	MVAINJ	4	BLS	AUTO ACC W/INJURIES	9,514	4,030	-5,484	-57.64
Trauma	CHILDA	6	BLS	CHILD ABUSE	39	17	-22	-56.41
Injury	PEDSTR	3	BLS	PEDESTRIAN STRUCK	4,645	2,372	-2,273	-48.93
Allergy	ANAPH	2	ALS	ANAPHYLAXIS	2,111	1,097	-1,014	-48.03
Obstetrics	GYNMAJ	3	BLS	GYN/SEVERE PAIN/BLEEDING	415	271	-144	-34.70
Obstetrics	GYNHEM	5	BLS	GYN BLEEDING/PT NOT PREGNANT	793	523	-270	-34.05
Trauma	AMPMAJ	3	BLS	AMPUTATION, ARM, HAND,LEG,FOOT	6	4	-2	-33.33
Alcohol Drugs	DRUG	4	BLS	HX DRUG OR ALCOHOL ABUSE	23,435	15,943	-7,492	-31.97
Cardiovascular	CVAC	2	BLS	STROKE	4,288	2,930	-1,358	-31.67
Other	JUMPDN	2	BLS	JUMPER DOWN	218	149	-69	-31.65
Abdominal Pain	ABDPN	5	BLS	ABDOMINAL PAIN	12,129	8,372	-3,757	-30.98
Obstetrics	OBCOMP	2	ALS	OBSTETRIC COMPLICATIONS	363	251	-112	-30.85
Neurological	SEIZR	3	BLS	SEIZURES	2,885	2,019	-866	-30.02
Allergy	MEDRXN	5	BLS	REACTION TO MEDICATION	544	383	-161	-29.60
Injury	INJURY	5	BLS	NON-CRITICAL INJURY	37,415	26,541	-10,874	-29.06
Neurological	STATEP	2	ALS	STATUS EPILEPTICUS	6,042	4,328	-1,714	-28.37
Respiratory	ASTHMB	2	BLS	ASTHMA ATTACK	2,955	2,135	-820	-27.75
Obstetrics	OBMAJ	2	BLS	MAJOR OBSTETRICAL COMPLAINT	1,140	824	-316	-27.72
Choking	CHOKE	1	DUAL	CHOKING	427	309	-118	-27.63
Infection	SICMIN	7	BLS	MINOR ILLNESS	4,243	3,087	-1,156	-27.24
Trauma	INBLED	3	ALS	INTERNAL BLEEDING	2,803	2,054	-749	-26.72
Respiratory	DIFFBR	2	ALS	DIFFICULT BREATHER	20,669	15,405	-5,264	-25.47
Obstetrics	OBLAB	5	BLS	FEMALE IN LABOR	1,379	1,035	-344	-24.95
Injury	INJMAJ	3	ALS	MAJOR INJURY	11,295	8,605	-2,690	-23.82
Obstetrics	OBMIS	4	BLS	MISCARRIAGE	686	524	-162	-23.62
Infection	SICK	6	BLS	SICK	51,298	39,654	-11,644	-22.70
Psych	EDP	7	BLS	PSYCHIATRIC PATIENT	19,959	15,670	-4,289	-21.49
Unconscious	UNC	2	ALS	UNCONSCIOUS PATIENT	15,897	12,751	-3,146	-19.79
Injury	INJMIN	7	BLS	MINOR INJURY	2,173	1,786	-387	-17.81
Trauma	TRAUMA	2	BLS	MULTIPLE TRAUMA PATIENT	1,147	943	-204	-17.79
Cardiovascular	CARD	3	ALS	CARDIAC CONDITION	15,160	12,507	-2,653	-17.50
Trauma	BURNMI	7	BLS	MINOR BURNS <18% ADLT OR <10%	270	225	-45	-16.67
Fire Police	ACTIVE	4	BLS	ACTIVE SHOOTER	384	321	-63	-16.41
Injury	MVA	6	BLS	AUTO ACCIDENT, NO CONFIRMD INJ	131	113	-18	-13.74
Cardiovascular	CVA	4	BLS	STROKE	1,424	1,285	-139	-9.76
Cardiovascular	CARDBR	3	ALS	CARDIAC CONDITION	17,450	16,001	-1,449	-8.30
Trauma	COLD	5	BLS	HYPOTHERMIA	43	40	-3	-6.98
Respiratory	INHALE	5	BLS	INHALATION OF SMOKE	264	247	-17	-6.44
Cardiovascular	HYPTN	5	BLS	HYPERTENSION	1,125	1,096	-29	-2.58
Psych	ALTMEN	3	ALS	ALTERED MENTAL STATUS	6,488	6,328	-160	-2.47
Injury	MVAINM	4	BLS	AUTO ACCIDENT, NO CONFIRMD INJ	3	0	-3	Decrease
Infection	SICKFT	6	BLS	SICK PATIENT FEVER\TRAVEL	1	0	-1	Decrease
Infection	SICKRF	6	BLS	SICK - RASH AND FEVER	1	0	-1	Decrease
Total	—	—	—	—	286,143	212,889	-73,254	-25.60%

[H]

Table 5: **FDNY Year/Year Overall Changes in Call Volume By Grouped Call Types, Priority, Ambulance Response and Description (March 20–June 13)**

Category	Call Types	Priority	Ambulance	Description	2019	2020	Change	(%) Change
Abdominal Pain	ABDPN	5	BLS	ABDOMINAL PAIN	12,129	8,372	-3,757	-30.98
Abdominal Pain	ABDPFC	5	BLS	ABDOMINAL PAIN FEVER COUGH	0	1,040	1,040	Increase
Alcohol Drugs	DRUGFC	4	BLS	HX DRUG OR ALCHL ABUSE FEV COU	0	334	334	Increase
Alcohol Drugs	DRUG	4	BLS	HX DRUG OR ALCOHOL ABUSE	23,435	15,943	-7,492	-31.97
Allergy	ANAPFC	2	ALS	ANAPHYLACTIC SHOCK FEVER COUGH	0	166	166	Increase
Allergy	ANAPH	2	ALS	ANAPHYLAXIS	2,111	1,097	-1,014	-48.03
Allergy	MEDRFC	5	BLS	REACTION TO MED FEVER COUGH	0	32	32	Increase
Allergy	MEDRXN	5	BLS	REACTION TO MEDICATION	544	383	-161	-29.60
Caller NotSpecific	UNKNOW	4	BLS	CALLER HAS NO PT MEDICAL INFO	14,875	16,995	2,120	14.25
Cardiovascular	ARREFC	1	DUAL	CARD OR RESP ARREST FEVER COUGH	0	1,346	1,346	Increase
Cardiovascular	ARREST	1	DUAL	CARDIAC ARREST	5,105	9,775	4,670	91.48
Cardiovascular	CVAC	2	BLS	STROKE	4,288	2,930	-1,358	-31.67
Cardiovascular	CVACFC	2	BLS	STROKE CRITICAL FEVER COUGH	0	298	298	Increase
Cardiovascular	CVAFC	2	BLS	STROKE FEVER COUGH	0	177	177	Increase
Cardiovascular	CARD	3	ALS	CARDIAC CONDITION	15,160	12,507	-2,653	-17.50
Cardiovascular	CARDBR	3	ALS	CARDIAC CONDITION	17,450	16,001	-1,449	-8.30
Cardiovascular	CARDFC	3	ALS	CARDIAC CONDITION FEVER COUGH	0	1,857	1,857	Increase
Cardiovascular	CVA	4	BLS	STROKE	1,424	1,285	-139	-9.76
Cardiovascular	HYPTN	5	BLS	HYPERTENSION	1,125	1,096	-29	-2.58
Choking	CHOKFC	1	DUAL	CHOKING FEVER COUGH	0	20	20	Increase
Choking	CHOKE	1	DUAL	CHOKING	427	309	-118	-27.63
Fire Police	ACTIVE	4	BLS	ACTIVE SHOOTER	384	321	-63	-16.41
Fire Police	PD13	7	BLS	POLICE	263	383	120	45.63
Infection	SICPED	4	BLS	SICK PEDIATRIC, <5 YEAR OLD	2,356	664	-1,692	-71.82
Infection	PEDFC	6	BLS	SICK PED <5 YRS FEVER COUGH	0	490	490	Increase
Infection	SICK	6	BLS	SICK	51,298	39,654	-11,644	-22.70
Infection	SICKFC	6	BLS	SICK COUGH FEVER	0	10,055	10,055	Increase
Infection	SICKFT	6	BLS	SICK PATIENT FEVER TRAVEL	1	0	-1	Decrease
Infection	SICKRF	6	BLS	SICK RASH AND FEVER	1	0	-1	Decrease
Infection	SICMFC	6	BLS	SICK COUGH FEVER	0	482	482	Increase
Infection	SICMIN	7	BLS	MINOR ILLNESS	4,243	3,087	-1,156	-27.24
Injury	VENOM	2	BLS	VENOM SNAKE BITES	2	3	1	50.00
Injury	INJMAJ	3	ALS	MAJOR INJURY	11,295	8,605	-2,690	-23.82
Injury	PEDSTR	3	BLS	PEDESTRIAN STRUCK	4,645	2,372	-2,273	-48.93
Injury	MVAINJ	4	BLS	AUTO ACC WITH INJURIES	9,514	4,030	-5,484	-57.64
Injury	MVAINM	4	BLS	AUTO ACCIDENT NO CONFIRMD INJ	3	0	-3	Decrease
Injury	INJURY	5	BLS	NON CRITICAL INJURY	37,415	26,541	-10,874	-29.06
Injury	MVA	6	BLS	AUTO ACCIDENT NO CONFIRMD INJ	131	113	-18	-13.74
Injury	INJMIN	7	BLS	MINOR INJURY	2,173	1,786	-387	-17.81

Table 5: **FDNY Year/Year Changes in Call Volume By Grouped Call Types, Priority, Ambulance Response *(continued)***

Category	Call Types	Priority	Ambulance	Description	Y2019	Y2020	Change	(%) Change
Neurological	STATFC	2	ALS	MULT OR PROLONG SEIZUR FEV COU	0	249	249	Increase
Neurological	STATEP	2	ALS	STATUS EPILEPTICUS	6,042	4,328	-1,714	-28.37
Neurological	SEIZFC	3	BLS	SEIZURES FEVER COUGH	0	129	129	Increase
Neurological	SEIZR	3	BLS	SEIZURES	2,885	2,019	-866	-30.02
Obstetrics	OBMAJ	2	BLS	MAJOR OBSTETRICAL COMPLAINT	1,140	824	-316	-27.72
Obstetrics	OBCOMP	2	ALS	OBSTETRIC COMPLICATIONS	363	251	-112	-30.85
Obstetrics	OBOUT	3	BLS	BABY OUT OR IMMINENT BIRTH	65	71	6	9.23
Obstetrics	GYNMAJ	3	BLS	GYN/SEVERE PAIN BLEEDING	415	271	-144	-34.70
Obstetrics	OBMIS	4	BLS	MISCARRIAGE	686	524	-162	-23.62
Obstetrics	OBLAB	5	BLS	FEMALE IN LABOR	1,379	1,035	-344	-24.95
Obstetrics	GYNHEM	5	BLS	GYN BLEEDING PT NOT PREGNANT	793	523	-270	-34.05
Other	JUMPDN	2	BLS	JUMPER DOWN	218	149	-69	-31.65
Other	JUMPUP	7	BLS	JUMPER UP	94	116	22	23.40
Psych	ALTMFC	3	ALS	ALT MENTAL STATUS FEVER COUGH	0	985	985	Increase
Psych	ALTMEN	3	ALS	ALTERED MENTAL STATUS	6,488	6,328	-160	-2.47
Psych	EDPC	7	BLS	PSYCHIATRIC PATIENT	6,453	7,925	1,472	22.81
Psych	EDP	7	BLS	PSYCHIATRIC PATIENT	19,959	15,670	-4,289	-21.49
Psych	EDPW	7	BLS	PSYCHIATRIC PATIENT	12	5	-7	-58.33
Respiratory	ASTHFC	2	BLS	ASTHMA ATTACK FEVER COUGH	0	758	758	Increase
Respiratory	DIFFFC	2	ALS	DIFF BREATHING FEVER COUGH	0	8,429	8,429	Increase
Respiratory	DIFFBR	2	ALS	DIFFICULT BREATHER	20,669	15,405	-5,264	-25.47
Respiratory	ASTHMB	2	BLS	ASTHMA ATTACK	2,955	2,135	-820	-27.75
Respiratory	RESPFC	4	BLS	RESP DISTRESS FEVER COUGH	0	2,035	2,035	Increase
Respiratory	RESPRF	4	BLS	RESPIRATORY DISTRESS	0	1	1	Increase
Respiratory	RESPIR	4	BLS	RESPIRATORY DISTRESS	3,565	8,268	4,703	131.92
Respiratory	INHALE	5	BLS	INHALATION OF SMOKE	264	247	-17	-6.44
Trauma	DROWN	2	DUAL	DROWNING	29	40	11	37.93
Trauma	TRAUMA	2	BLS	MULTIPLE TRAUMA PATIENT	1,147	943	-204	-17.79
Trauma	INBLFC	3	ALS	INTERNAL BLEEDING FEVER COUGH	0	337	337	Increase
Trauma	SHOT	3	BLS	GUN SHOT WOUND	213	268	55	25.82
Trauma	ELECT	3	ALS	ELECTROCUTION	54	60	6	11.11
Trauma	BURNMA	3	ALS	MAJOR BURNS 18Pct ADLT 10Pct CHILD	239	248	9	3.77
Trauma	STAB	3	BLS	STABBING	963	983	20	2.08
Trauma	INBLED	3	ALS	INTERNAL BLEEDING	2,803	2,054	-749	-26.72
Trauma	AMPMAJ	3	BLS	AMPUTATION ARM HAND LEG FOOT	6	4	-2	-33.33
Trauma	AMPMIN	3	BLS	AMPUTATION FINGERS OR TOES	76	32	-44	-57.89
Trauma	HEAT	4	BLS	HEAT EXHAUSTION	42	13	-29	-69.05
Trauma	COLD	5	BLS	HYPOTHERMIA	43	40	-3	-6.98
Trauma	CHILDA	6	BLS	CHILD ABUSE	39	17	-22	-56.41
Trauma	BURNMI	7	BLS	MINOR BURNS $<$18% ADLT OR $<$10%	270	225	-45	-16.67
Unconscious	UNCFC	2	ALS	UNC PATIENT FEVER COUGH	0	986	986	Increase
Unconscious	UNC	2	ALS	UNCONSCIOUS PATIENT	15,897	12,751	-3,146	-19.79
Unknown Cond	OTHER	6	BLS	UNKNOWN CONDITION	1,688	3,295	1,607	95.20
Total	—	—	—	—	319,751	291,525	-28,226	-8.83

Call types such as EVAC, EVENT, Standby, Transfer and NA are excluded,
Disposition codes 86, 87, 90 and NA are excluded and Priority codes 8 and 9 are excluded.
